# Voriconazole or Amphotericin B as Primary Therapy Yields Distinct Early Serum Galactomannan Trends Related to Outcomes in Invasive Aspergillosis

**DOI:** 10.1371/journal.pone.0090176

**Published:** 2014-02-28

**Authors:** Louis Yi Ann Chai, Bart Jan Kullberg, Arul Earnest, Elizabeth M. Johnson, Steven Teerenstra, Alieke G. Vonk, Haran T. Schlamm, Raoul Herbrecht, Mihai G. Netea, Peter F. Troke

**Affiliations:** 1 Department of Medicine, Radboud University Nijmegen Medical Centre, Nijmegen, the Netherlands; 2 Nijmegen Institute for Infection, Inflammation and Immunity (N4i), Nijmegen, the Netherlands; 3 Division of Infectious Diseases, University Medicine Cluster, National University Health System, Singapore, Singapore; 4 Center for Quantitative Medicine, Duke-NUS Graduate Medical School, Singapore, Singapore; 5 Mycology Reference Laboratory, Health Protection Agency, Bristol, United Kingdom; 6 Department of Epidemiology, Biostatistics and Health Technology Assessment, Radboud University Nijmegen Medical Centre, Nijmegen, the Netherlands; 7 Department of Medical Microbiology and Infectious Diseases, Erasmus MC, University Medical Centre Rotterdam, Rotterdam, the Netherlands; 8 Pfizer Global Research and Development, New York, New York, United States of America; 9 Department of Oncology and Hematology, Hôpitaux Universitaires de Strasbourg, Strasbourg, France; 10 The Old Court, Broadstairs, Kent, United Kingdom; Leibniz Institute for Natural Products Research and Infection Biology- Hans Knoell Institute, Germany

## Abstract

An improved number of anti-fungal drugs are currently available for the treatment of invasive aspergillosis (IA). While serial galactomannan index (GMI) measurement can be used to monitor response to treatment, the extent to which different anti-fungal regimens can affect galactomannan levels is unknown. In 147 IA patients receiving either voriconazole (VCZ) or conventional amphotericin B (CAB) in a multicentre clinical trial, we performed post-hoc analyses of GMI trends in relation to outcomes. The generalized estimation equations approach was used to estimate changes in the effect size for GMI over time within patients. Patients who received VCZ primary therapy and had good treatment response 12 weeks later showed earlier decreases in GMI values at Week 1 and Week 2 (p = 0.001 and 0.046 respectively) as compared to patients who only received CAB. At end-of-randomized therapy (EORT), which was a pre-set secondary assessment point for all patients who switched from randomized primary (CAB or VCZ) to an alternative anti-fungal drug, treatment failure was associated with increasing GMI at Weeks 1 and 2 in CAB-primary treated patients (p = 0.022 and 0.046 respectively). These distinct trends highlight the variations in GMI kinetics with the use of different anti-fungal drugs and their implications in relation to IA treatment response.

## Introduction

Invasive aspergillosis (IA) is the most common opportunistic mold infection in immunocompromised patients and leads to significant morbidity and mortality. The presence of neutropenia and the concurrent use immunomodulatory agents not only hinder the ability of the host to mount an efficient inflammatory response against the pathogen but also make the monitoring of response to anti-fungal treatment difficult. Galactomannan is a distinct polysaccharide component of the *Aspergillus* cell wall and its quantification can serve as a surrogate marker for fungal burden [1]. The detection of galactomannan with the Platelia *Aspergillus* EIA, the galactomannan index (GMI) has been adopted as a criterion in the diagnosis of IA [2], [3] and it has also been suggested that serial determination of serum GMI may be useful for monitoring the response to treatment [4]. Recently, we explored the prognostic usefulness of serial GMI measurements and reported that early GMI trends at Week 1 and Week 2 of anti-fungal treatment were of value for predicting eventual clinical outcomes [5]. Our analysis was based on the well-characterized IA patient cohort in the landmark multicenter Global Aspergillosis Study, which compared the efficacy of voriconazole (VCZ) versus conventional amphotericin B deoxycholate (CAB) as primary therapy for IA [6]. However the influence of the respective anti-fungal agents on GMI trends was not delineated in the above study.

Voriconazole has since become the recommended primary drug of choice for treatment of IA [7]. However, alternative anti-fungal regimens, including amphotericin-based formulations and the echinocandins, continue to be prescribed for IA patients when voriconazole use is contraindicated due to intolerance, or in the setting of azole-resistant *Aspergillus* species [8]. Furthermore, in those regions where healthcare resources remain restricted, amphotericin B deoxycholate continues to be a commonly used anti-fungal agent [9]. The respective therapies may exhibit differing efficacies and may also differentially influence GMI measurements. This could have a critical bearing on the interpretation of serial GMI trends in relation to clinical response. However in this context, the effect of anti-fungal regimens on GMI trends during treatment has never been studied.

Our hypothesis was that anti-fungal drugs could have distinct influences on early GMI trends that may subsequently predict clinical outcome. We tested this hypothesis, post-hoc, in a well-characterized cohort of study patients who received either VCZ or CAB for the primary treatment of IA.

## Patients and Methods

The patients studied were from Protocol 150–307 of the Global Comparative Aspergillosis Study, a multicenter randomized trial conducted in Europe, Australia and Israel that compared the efficacy of VCZ versus CAB for the primary treatment of IA. The lead organiser of the study was the European Organisation for Research and Treatment of Cancer (EORTC) (with the respective protocol identifiers EORTC-19961 and ClinicalTrials.gov NCT00003031). The protocol was approved by the appropriate institutional review boards in all participating centers, and written informed consent was obtained from all patients. The selection of patients and conduct of the trial were as previously published [6]. Patients assessed by the attending physician as having no response or intolerance to the initial randomized drug (VCZ or CAB) could be switched to other licensed antifungal therapy (OLAT). Blood specimens were obtained serially from trial patients at baseline, upon recruitment, and at Weeks 1, 2 and 4 following commencement of randomized anti-fungal therapy. The current study was a post-hoc analysis of serial serum GM trends of patients receiving anti-fungal treatment for IA. Serum GM was measured at a central laboratory (Health Protection Agency Mycology Reference Laboratory, Bristol, U.K.) within 2 years upon completion of the trial and was performed according to the manufacturer's instructions (Platelia *Aspergillus* EIA, Bio-Rad Laboratories, Marne-la-Coquette, France). Random testing for stability of the stored samples showed an average GMI decrease of 11% over 13 months. Acute kidney injury (AKI) was defined as a 2-fold increase in serum creatinine during anti-fungal therapy in relation to the baseline level before starting treatment, or a level >3 mg/dL (265 µmol/L) if the baseline value was >1.5 mg/dL (133 µmol/L) [6].

Outcome measures were assessed by an independent and blinded data review committee (DRC) based on reviews of clinical, mycological and systematically-collected radiological data. As the primary end-point, satisfactory clinical response was defined as a complete or partial response at Week 12 after commencement of anti-fungal therapy (W12 Response); poor response was defined as treatment failure or stable disease (W12 Non-response), in accordance with the pre-established assessment criteria of the DRC. In cases whereby death had occurred prior to the stipulated DRC assessment time-point or that the clinical data was insufficient to determine an objective response, the DRC W12 Response was set as ‘indeterminate’. End-of-initial randomized therapy (EORT) was a secondary endpoint reached when pre-specified criteria to stop the randomized therapy and switch to OLAT were attained. In patients who remained on their initial randomized therapy until week 12, the EORT assessment and the Week 12 assessment were the same. Post-hoc assessment of the clinical response at EORT by the DRC (*v.i.z.* satisfactory, unsatisfactory or indeterminate) used the same criteria as for the W12 Response. The other definitive outcome measure was all-cause survival at 12 weeks after the start of anti-fungal treatment.

In our analysis, only the GMI of patients who continued to receive the primary randomized anti-fungal agent (CAB or VCZ) at the specified intervals of therapy (v.i.z. Weeks 1, 2 and 4) were considered. Patients who were switched from the primary trial drug to OLAT were dropped from the analysis at the point of drug switch. The time profile of GMI or ΔGMI (GMI-change between 2 specified time-points) for the different outcomes at week 12 (based on clinical response and survival at week 12) are presented graphically by plotting the mean per time-point. The generalized estimation equations (GEE) approach is used to test and estimate changes in the effect size for GMI, accounting for repeated measurements over time within patients. A Gaussian family distribution is specified along with an identity link function and an exchangeable correlation structure. Where relevant, sensitivity analysis incorporating renal impairment as variable was performed. All statistical evaluations were performed using STATA release 12.0 (StataCorp, College Station, TX). The threshold for statistical significance is set at p<0.05.

## Results

Data from one hundred and forty-seven patients with proven and probable IA [2] and who had GMI measurements performed were studied, based on the modified intention-to-treat analysis [6]. Of these patients, 77 were randomized to receive CAB, while 70 received VCZ as primary therapy. The demographics of these study patients are as described in Table 1. Treatment success at W12 for patients who received CAB as primary therapy was 37.7% as compared to 55.7% for VCZ. Treatment success at the EORT assessment for patients who were randomized to receive CAB was 18.3% versus 54.3% for those randomized to VCZ (p<0.001). Survival at end of study (at Week 12) was 66.2% for patients initially randomized to CAB and 74.3% for patients initially randomized to VCZ.

**Table 1:** Demographics characteristics of study cohort of 147 patients.

Characteristics	Patients (n = 147)		P value
	*CAB N = 77 (%)*	*VCZ N = 70 (%)*	
***Sex***			
**Male**	51 (66.2)	48 (68.6)	0.861
**Female**	26 (33.8)	22 (31.4)	
***Certainty of Disease***			
**Proven IA**	8 (10.4)	9 (12.8)	0.797
**Probable IA**	69 (89.6)	61 (87.2)	
***Neutropenia***	45 (58.4)	44 (62.9)	0.353
***Underlying Disease***			
**Leukaemia/Lymphoma**	59 (76.6)	45 (64.3)	0.526
**HSCT**	14 (18.2)	19 (27.1)	
***Week 12 Response***			
**Satisfactory**	29 (37.7)	39 (55.7)	
**Unsatisfactory**	15 (19.4)	10 (14.3)	0.09
**Indeterminate**	33 (42.9)	21 (30.0)	
***EORT Response***			
**Satisfactory**	14 (18.2)	38 (54.3)	
**Unsatisfactory**	55 (71.4)	19 (27.1)	0.001
**Indeterminate**	8 (10.4)	13 (18.6)	
***Week 12 Survival***			
**Alive**	51 (66.2)	52 (74.3)	0.368
**Dead**	26 (33.8)	18 (25.7)	

CAB: conventional amphotericin B deoxycholate. VCZ: voriconazole. IA: invasive aspergillosis. HSCT: hematopoietic stem cell transplantation. EORT Response: End-of-initial randomized therapy response. The comparison of demographic and clinical characteristics between patients who received CAB or VCZ was made using the chi-squared or ANOVA test as appropriate.

**Figure 1:**
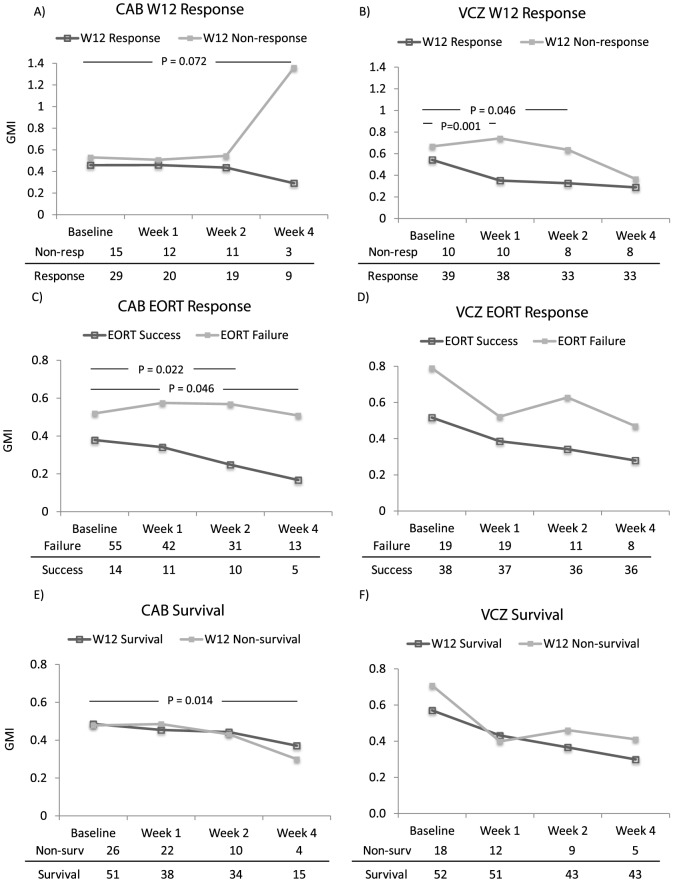
A–F Galactomannan index (GMI) trends of patients receiving as primary therapy, either conventional amphotericin B (CAB, Panels A,C,E) or voriconazole (VCZ, Panels B,D,F) in relation to the respective outcomes. Geometric GMI (y-axis) means was plotted over indicated time intervals. Only the GMI of patients who continued to receive the primary randomized anti-fungal agent (CAB or VCZ) at the specified intervals of therapy were considered. Patients who were switched from the primary trial drug to OLAT were dropped from the analysis at the point of drug switch. Table below each Panel indicates the number of patients still receiving the respective primary antifungal therapy (*v.i.z.* CAB or VCZ) over time in each arm. The P value is indicative of the difference in the ΔGMIs between the two outcome-stratified arms from Baseline to the indicated study interval (v.i.z. Weeks 1, 2 or 4) as enclosed by the horizontal bars.

Serial GMI trends in relation to the specified outcomes (satisfactory or unsatisfactory response) were studied categorically between patients who received either VCZ or CAB as primary therapy (Figures 1A–F). In these analyses, patients with an ‘indeterminate’ response, as assessed by the DRC, were excluded; thus, only those with a DRC-determined non-response at either Week 12 or EORT were assessed and compared with treated patients who had responded to therapy (Figures 1A–D). We also performed analyses that included the ‘indeterminate’ response cases (but classed them as ‘non-response’) at either Week 12 or EORT and obtained similar results (data not shown).

Patients who were randomized to VCZ and had a satisfactory response at Wk12 showed a decreasing GMI at Week 1 and Week 2 relative to the GMI at baseline, as compared to those who eventually failed treatment (p = 0.001 and p = 0.046 respectively, Figure 1B). However, for patients randomized to receive CAB, this early difference in GMI trend between W12 responders and non-responders was delayed and not evident until Week 4 (Figure 1A).

At EORT, patients randomized to receive CAB (Figure 1C) and assessed not to have responded to treatment showed persistently elevated Baseline-to-Week 2 and Baseline-to-Week 4 GMI relative to patients who responded to CAB (p = 0.022 and p = 0.046 respectively). In VCZ patients, there was no difference in GMI trends between those who had a satisfactory or unsatisfactory EORT response (Figure 1D).

For both antifungal drugs, initial GMI trends (between Baseline and Week 2) were generally not prognostic for eventual survival in this analysis. Notably, however, in patients randomized to CAB, those who died before Week 12 showed a significantly greater Baseline-to-Week 4 decrease in GMI as compared to the Week 12 survivors (Figure 1E).

## Discussion

GMI quantification has been proposed as a surrogate for fungal burden [1] and treatment response [4]. In this study we show that temporal galactomannan trends are distinct in patients receiving different antifungal treatment regimens. In IA patients who received VCZ as primary therapy, a significant drop in GMI from baseline to Week 1 was prognostic for a satisfactory clinical response at Week 12, while persistence of an elevated GMI was associated with eventual treatment failure (Figure 1B). However, GMI trends suggesting failure on CAB treatment were seen later, at Week 4 of therapy (Figure 1A). In summary, the distinction between responders and non-responders was seen earlier in patients on primary VCZ treatment than in those receiving CAB. Progressive reduction of GMI however, was not a surrogate for eventual survival.

Quantification of fungal burden in animal models treated with either VCZ or CAB has shown that the former was more effective in clearance of *Aspergillus* with reduced tissue colony counts [10], from which galactomannan might be a surrogate for fungal load [1]. Interestingly, in the VCZ primary treatment arm, the promptness of response as reflected by clear GMI reduction within the 1^st^ 2 weeks of therapy was associated with a satisfactory response at Week 12. Early persistence of elevated GMI, on the other hand, may well indicate significant residual fungal burden as a result of slow response to treatment (Figures 1A–B). In contrast, in patients receiving CAB as primary therapy, prediction of eventual the response using GMI as surrogate was only possible at Week 4.

In CAB-treated patients, a persistently high (or unchanged) GMI from baseline to Week 2 or Week 4 (Figure 1C) was linked to the likelihood of switching earlier than VCZ to another antifungal therapy at EORT. However, it is notable that only a minority (4 patients) had switched from CAB to OLAT on the grounds of poor primary treatment response as assessed by the primary physician. The major indication for the switch to OLAT was drug (especially renal/electrolyte) intolerance [11]. CAB-associated intolerance is thus a potential confounder that may herald an unsatisfactory IA response to primary CAB treatment.

The capacity of amphotericin B to alter renal function is well recognized and galactomannan levels are partly determined by glomerular filtration rate, as it is also detectable in urine. As such, the delayed changes in serial GMI observed in patients receiving CAB, as compared to VCZ, might be influenced by differing rates of renal clearance of circulating galactomannan. Indeed clinically, the dissociation between GMI and IA treatment response in the setting of renal failure has already been described [12]. However, the extent to which amphotericin B and the development of acute kidney injury affect the kinetics of renal clearance over time is not well understood. To address this aspect in the patients who had received CAB as primary therapy, we further incorporated AKI as a variable in our analyses. The observed GMI trend differences between responders and non-responders at EORT remained largely unchanged (Baseline-to-Week 2 GMI change p = 0.022 became 0.026, and Baseline-to-Week 4 GMI change p = 0.046 became 0.064 respectively). The Baseline-to Week 4 GMI trend difference between W12 survivors and non-survivors (p = 0.014) became p = 0.05 with AKI as a co-variate. The above analyses suggested that renal impairment in patients receiving CAB did not influence GMI trend results to a significant extent.

Alternatively, these differences in GMI trends could be related to the mode of action of both drugs. As a polyene, amphotericin B acts principally through binding to ergosterol in the fungal plasma membrane and creating transmembrane pores which induce cell permeability and eventually death [13]. Voriconazole, a triazole, inhibits ergosterol synthesis through P450 cytochrome-mediated lanosterol demethylation [14]. Galactomannan, on the other hand, is a polysaccharide moiety within the *Aspergillus* cell wall lying exterior to the cell membrane [15]. So while both drugs exert their effects at the level of the cell membrane, observations have been made that azoles and polyenes can induce different changes to the fungal cell wall structure and composition [16]–[18]. Thus, they may also differentially influence the galactomannan content and shedding.

The statistical difference in the GMI trend for survival in CAB-treated subjects is unexpected. The GMI drop between Baseline and Week 4 for CAB seems linked to the likelihood of mortality at 12 weeks. This was discordant in relation to the GMI trends at Week 12 or at the EORT Response described above. This may be a chance occurrence, as differences in the magnitude of change between the survivors and non-survivors, while statistically significant, were small (Figure 1E). This is further mitigated by an observation in similar IA patients (of which the study cohort here is a subset) that mortality beyond 6 weeks of IA diagnosis and treatment was more likely related to the status of underlying primary disease than the effectiveness of anti-fungal treatment [19]. Nonetheless, it remains to be well established whether circulating galactomannan levels can be influenced by other important factors, such as the patient's primary disease and concurrent infections. Nonetheless concern that piperacillin-tazobactam usage might have significantly confounded GMI level is largely unfounded as this antibiotic saw its approval for use towards the end of the clinical trial (for Protocol 130–307 outside the US).

In conclusion, we demonstrate here that different anti-fungal treatments yield distinct early GMI trends that may have prognostic value. The increasing number of different classes of anti-fungals currently in use against IA highlights the need to further understand the variety of disease responses with different drugs classes and the kinetics of biomarker monitoring. Our findings illustrate this point and have potentially significant implications for the management of IA.

## References

[1] Musher B, Fredricks D, Leisenring W, Balajee SA, Smith C, et al (2004) Aspergillus galactomannan enzyme immunoassay and quantitative PCR for diagnosis of invasive aspergillosis with bronchoalveolar lavage fluid. J Clin Microbiol 42: 5517–5522. 15583275 10.1128/JCM.42.12.5517-5522.2004 PMC535238

[2] De Pauw B, Walsh TJ, Donnelly JP, Stevens DA, Edwards JE, et al (2008) Revised definitions of invasive fungal disease from the European Organization for Research and Treatment of Cancer/Invasive Fungal Infections Cooperative Group and the National Institute of Allergy and Infectious Diseases Mycoses Study Group (EORTC/MSG) Consensus Group. Clin Infect Dis 46: 1813–1821. 18462102 10.1086/588660 PMC2671227

[3] Maertens JA, Klont R, Masson C, Theunissen K, Meersseman W, et al (2007) Optimization of the cutoff value for the Aspergillus double-sandwich enzyme immunoassay. Clin Infect Dis 44: 1329–1336. 17443470 10.1086/514349

[4] Boutboul F, Alberti C, Leblanc T, Sulahian A, Gluckman E, et al (2002) Invasive aspergillosis in allogeneic stem cell transplant recipients: increasing antigenemia is associated with progressive disease. Clin Infect Dis 34: 939–943. 11880959 10.1086/339324

[5] Chai LY, Kullberg BJ, Johnson EM, Teerenstra S, Khin LW, et al (2010) Early serum galactomannan trend as a predictor of outcome of invasive aspergillosis. J Clin Microbiol 50: 2330–2336. 10.1128/JCM.06513-11 PMC3405588 22553232

[6] Herbrecht R, Denning DW, Patterson TF, Bennett JE, Greene RE, et al (2002) Voriconazole versus amphotericin B for primary therapy of invasive aspergillosis. N Engl J Med 347: 408–415. 12167683 10.1056/NEJMoa020191

[7] Walsh TJ, Anaissie EJ, Denning DW, Herbrecht R, Kontoyiannis DP, et al (2008) Treatment of aspergillosis: clinical practice guidelines of the Infectious Diseases Society of America. Clin Infect Dis 46: 327–360. 18177225 10.1086/525258

[8] Snelders E, van der Lee HA, Kuijpers J, Rijs AJ, Varga J, et al (2008) Emergence of azole resistance in Aspergillus fumigatus and spread of a single resistance mechanism. PLoS Med 5: e219. 18998768 10.1371/journal.pmed.0050219 PMC2581623

[9] Chakrabarti A, Chatterjee SS, Das A, Shivaprakash MR (2011) Invasive aspergillosis in developing countries. Med Mycol 49 Suppl 1S35–47. 20718613 10.3109/13693786.2010.505206

[10] Kirkpatrick WR, McAtee RK, Fothergill AW, Rinaldi MG, Patterson TF (2000) Efficacy of voriconazole in a guinea pig model of disseminated invasive aspergillosis. Antimicrob Agents Chemother 44: 2865–2868. 10991875 10.1128/aac.44.10.2865-2868.2000 PMC90166

[11] Patterson TF, Boucher HW, Herbrecht R, Denning DW, Lortholary O, et al (2005) Strategy of following voriconazole versus amphotericin B therapy with other licensed antifungal therapy for primary treatment of invasive aspergillosis: impact of other therapies on outcome. Clin Infect Dis 41: 1448–1452. 16231256 10.1086/497126

[12] El Saleeby CM, Allison KJ, Knapp KM, Walsh TJ, Hayden RT (2005) Discordant rise in galactomannan antigenemia in a patient with resolving Aspergillosis, renal failure, and ongoing hemodialysis. J Clin Microbiol 43: 3560–3563. 16000507 10.1128/JCM.43.7.3560-3563.2005 PMC1169100

[13] Czub J, Baginski M (2006) Modulation of amphotericin B membrane interaction by cholesterol and ergosterol–a molecular dynamics study. J Phys Chem 110: 16743–16753. 10.1021/jp061916g 16913814

[14] Sanati H, Belanger P, Fratti R, Ghannoum M (1994) A new triazole, voriconazole (UK-109,496), blocks sterol biosynthesis in Candida albicans and Candida krusei. Antimicrob Agents Chemother 41: 2492–2496. 10.1128/aac.41.11.2492 PMC164150 9371355

[15] Latge JP (2010) Tasting the fungal cell wall. Cell Microbiol 12: 863–872. 20482553 10.1111/j.1462-5822.2010.01474.x

[16] Belanger P, Nast CC, Fratti R, Sanati H, Ghannoum M (1997) Voriconazole (UK-109,496) inhibits the growth and alters the morphology of fluconazole-susceptible and -resistant Candida species. Antimicrob Agents Chemother 41: 1840–1842. 9257776 10.1128/aac.41.8.1840 PMC164020

[17] da Silva Ferreira ME, Malavazi I, Savoldi M, Brakhage AA, Goldman MH, et al (2006) Transcriptome analysis of Aspergillus fumigatus exposed to voriconazole. Curr Genet 50: 32–44. 16622700 10.1007/s00294-006-0073-2

[18] Gautam P, Shankar J, Madan T, Sirdeshmukh R, Sundaram CS, et al (2008) Proteomic and transcriptomic analysis of Aspergillus fumigatus on exposure to amphotericin B. Antimicrob Agents Chemother 52: 4220–4227. 18838595 10.1128/AAC.01431-07 PMC2592866

[19] Wingard JR, Ribaud P, Schlamm HT, Herbrecht R (2008) Changes in causes of death over time after treatment for invasive aspergillosis. Cancer 112: 2309–2312. 18338758 10.1002/cncr.23441

